# Long non-coding RNAs *ENST00000429730.1* and *MSTRG.93125.4* are associated with metabolic activity in tuberculosis lesions of sputum-negative tuberculosis patients

**DOI:** 10.18632/aging.202634

**Published:** 2021-03-03

**Authors:** Lin Wang, Zilu Wen, Hui Ma, Liwei Wu, Hui Chen, Yijun Zhu, Liangfei Niu, Qihang Wu, Hongwei Li, Lei Shi, Leilei Li, Leiyi Wan, Jun Wang, Ka-Wing Wong, Yanzheng Song

**Affiliations:** 1 Department of Thoracic Surgery, Shanghai Public Health Clinical Center, Fudan University, Shanghai, China; 2 Department of Scientific Research, Shanghai Public Health Clinical Center, Fudan University, Shanghai, China

**Keywords:** sputum-negative tuberculosis, RNA-sequencing, lncRNA

## Abstract

Accurate diagnosis of complete inactivation of tuberculosis lesions is still a challenge with respect to sputum-negative tuberculosis. RNA-sequencing was conducted to uncover potential lncRNA indicators of metabolic activity in tuberculosis lesions. Lung tissues with high metabolic activity and low metabolic activity demonstrated by fluorine-18-fluorodeoxyglucose positron emission tomography/computed tomography were collected from five sputum-negative tuberculosis patients for RNA-sequencing. Differentially-expressed mRNAs and lncRNAs were identified. Their correlations were evaluated to construct lncRNA-mRNA co-expression network, in which lncRNAs and mRNAs with high degrees were confirmed by quantitative real-time PCR using samples collected from 11 patients. Prediction efficiencies of lncRNA indicators were assessed by receiver operating characteristic curve analysis. Bioinformatics analysis was performed for potential lncRNAs. 386 mRNAs and 44 lncRNAs were identified to be differentially expressed. Differentially-expressed mRNAs in lncRNA-mRNA co-expression network were significantly associated with fibrillar collagen, platelet-derived growth factor binding, and leukocyte migration involved in inflammatory response. Seven mRNAs (*C1QB*, *CD68*, *CCL5*, *CCL19*, *MMP7*, *HLA-DMB*, and *CYBB*) and two lncRNAs (*ENST00000429730.1* and *MSTRG.93125.4*) were validated to be significantly up-regulated. The area under the curve of *ENST00000429730.1* and *MSTRG.93125.4* was 0.750 and 0.813, respectively. Two lncRNAs *ENST00000429730.1* and *MSTRG.93125.4* might be considered as potential indicators of metabolic activity in tuberculosis lesions for sputum-negative tuberculosis.

## INTRODUCTION

Tuberculosis is one of the major public health problems globally. More than 1.7 billion people, that is, about 25% of the world population, are estimated to be infected with *Mycobacterium tuberculosis* (*M. tuberculosis*) [1]. According to the World Health Organization, around 10 million individuals fall ill with tuberculosis each year and 1.6 million die [2]. Successful treatment of pulmonary tuberculosis largely depends on making an accurate diagnosis and starting standardized anti-tuberculosis treatment. In clinical practice, tuberculosis is treated based on patient symptoms, chest radiography (chest X-ray) abnormalities and sputum bacteriological examination. Sputum smear test can find acid-fast bacilli in almost 50-60% of pulmonary tuberculosis [3]. However, in some cases of current active pulmonary tuberculosis, neither bacteriological examination nor serial chest X-ray unequivocally demonstrates the activity of disease. Radiographic findings suggested that the sputum-negative pulmonary tuberculosis without clinical or microbial evidence is one of the most dangerous factors for the development of active tuberculosis [4].

Although smear-negative tuberculosis patients have lower risk of spreading tuberculosis than the smear-positive patients, smear-negative tuberculosis patients are still able to transmit tuberculosis. The relative transmission rate of smear-negative tuberculosis patients in comparison with smear-positive tuberculosis patients has been ascertained at 22% base on a molecular epidemiologic method [5]. Nevertheless, nearly 50% patients with tuberculosis are sputum smear-negative cases, as a result of which the total contribution of smear-negative tuberculosis patients to the transmission of tuberculosis is notable. Polymerase chain response (PCR) can quickly analyze sputum samples, while its sensitivity is low [6]. As a result, clinicians often hesitate in starting anti-tuberculosis treatment for fear of the potential side-effects of anti-tuberculosis drugs. However, some patients received a standard 6-month anti-tuberculosis treatment with- smear and culture turned negative, while the lungs of those patients were remained lesions. About 5% of patients with drug-sensitive pulmonary tuberculosis will experience relapse within six months of completing treatment with a standard 6-month anti-tuberculosis treatment [7, 8]. Tuberculosis relapse is caused by persisting slow growing, metabolically active but non-culturable bacilli that are less sensitive to chemotherapy agents [9, 10]. In cases of prolonged therapy, treatment outcome is difficult to be classified as a success as negative sputum smear is only an indicator for contagiousness but not for disease activity [11].

Positron emission tomography with computed tomography (PET/CT) using fluorine-18-fluorodeoxyglucose (FDG) is a useful imaging modality for guiding management of oncology patients [12]. FDG, an analog of glucose, accumulates at the bacterial infection sites, such as tuberculous granuloma, due to the high consumption of glucose by activated macrophages, the most abundant inflammatory cell in the granuloma [13]. Accumulation of FDG in tuberculosis lesions has been shown to correlate with the number of bacilli and a higher risk of recurrence of hypermetabolic lesions [14, 15]. Although high uptake in pulmonary tuberculosis lesions suggests active disease, it may also indicate a host immune system response that will eventually prevail. However, the immune response is more active in hyper metabolized lesions on PET. When immunosuppression occurs in the host, if the lesion contains residual *M. tuberculosis*, it is more likely to evolve into recurrent tuberculosis [16]. However, FDG-PET/CT is too expensive to be used as an indicator of complete inactivity of tuberculosis lesions. Therefore, it is urgently needed to uncover biological indicators for accurate diagnosis of complete inactivation of tuberculosis lesions, which may provide proof for a complete cure of tuberculosis in the future.

Long non-coding RNAs (lncRNAs) are molecules that could regulate the expression of protein-coding genes to participate in various cellular events. Dysregulations of lncRNAs have been found associated with many cancers and infectious diseases [17]. In this present study, lung tissue samples with low metabolic activity and high metabolic activity according to FDG-PET/CT were collected from patients with sputum-negative pulmonary tuberculosis. The potential lncRNA biomarkers that can be used as indicators for a complete cure of tuberculosis were screened out by sequencing and bioinformatics methods.

## RESULTS

### Differentially expressed mRNAs and differentially expressed lncRNAs

**Figure 1:**
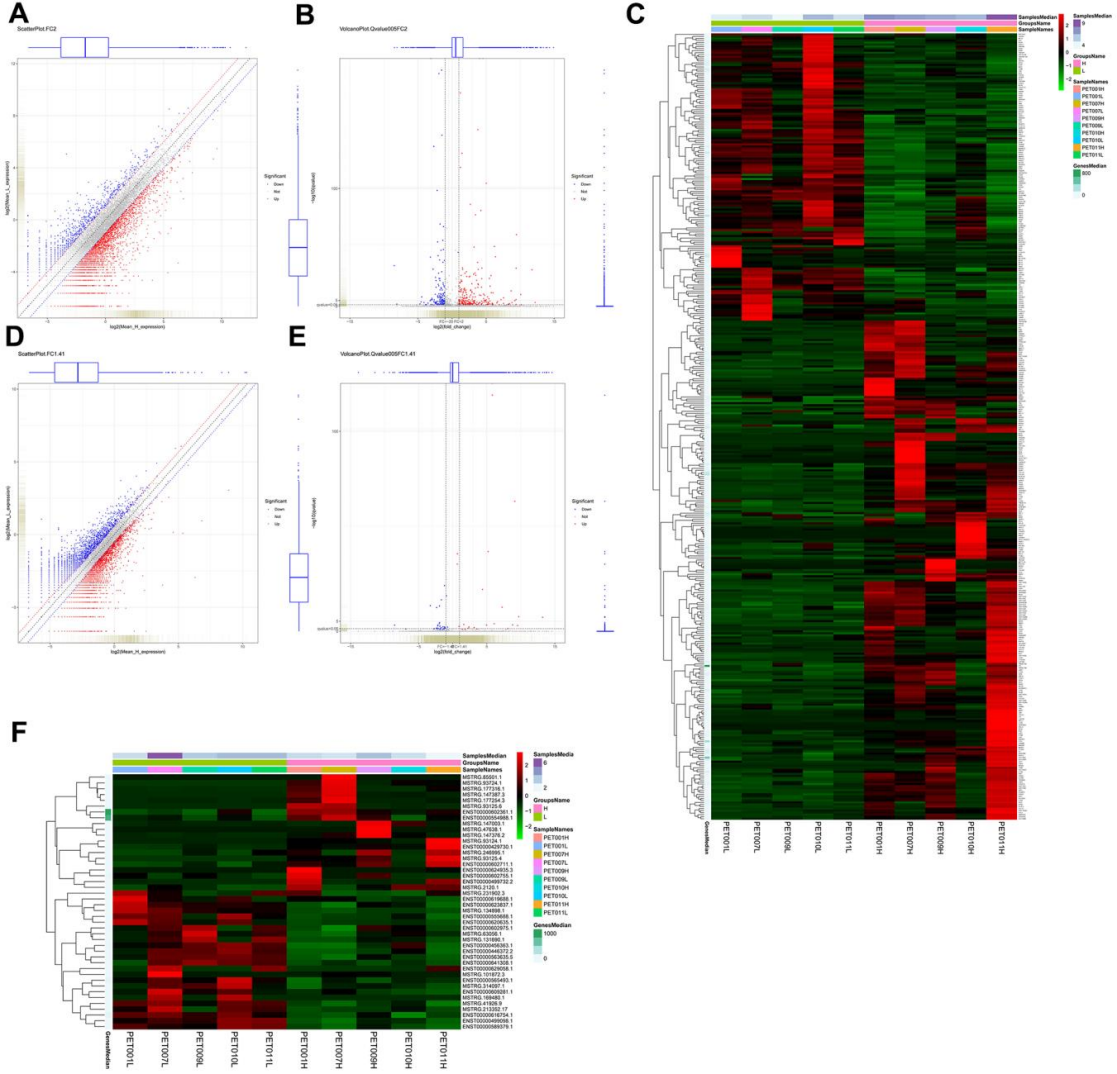
**Identification and hierarchical clustering analysis of differentially expressed mRNAs and lncRNAs between lung tissue samples with high metabolic activity (PET-high) and lung tissue samples with low metabolic activity (PET-low).** (**A**) scatter plot of mRNAs with |log_2_ fold change (FC) | > 1; (**B**) volcano plot of mRNAs with |log_2_FC | > 1 (FC > 2) and q value < 0.05; (**C**) Hierarchical clustering heatmap of differentially expressed mRNAs; (**D**) scatter plot of lncRNAs with |log_2_ FC | > 0.5 (FC > 1.41); (**E**) volcano plot of lncRNAs with |log_2_ FC | > 0.5 and q value < 0.05; (**F**) hierarchical clustering heatmap of differentially expressed lncRNAs.

386 mRNAs (141 down-regulated and 245 up-regulated mRNAs) were differentially expressed in PET-high samples when compared to PET-low samples (Figure 1A, 1B). Hierarchical clustering of these differentially expressed mRNAs indicated that PET-high samples were indeed distinct from PET-low samples (Figure 1C). Moreover, 44 differentially expressed lncRNAs (24 down-regulated and 20 up-regulated lncRNAs) were identified between PET-high and PET-low samples (Figure 1D, 1E). Hierarchical clustering of differentially expressed lncRNAs again revealed that PET-high and PET-low samples were clustered in two distinct groups (Figure 1F).

**Figure 2:**
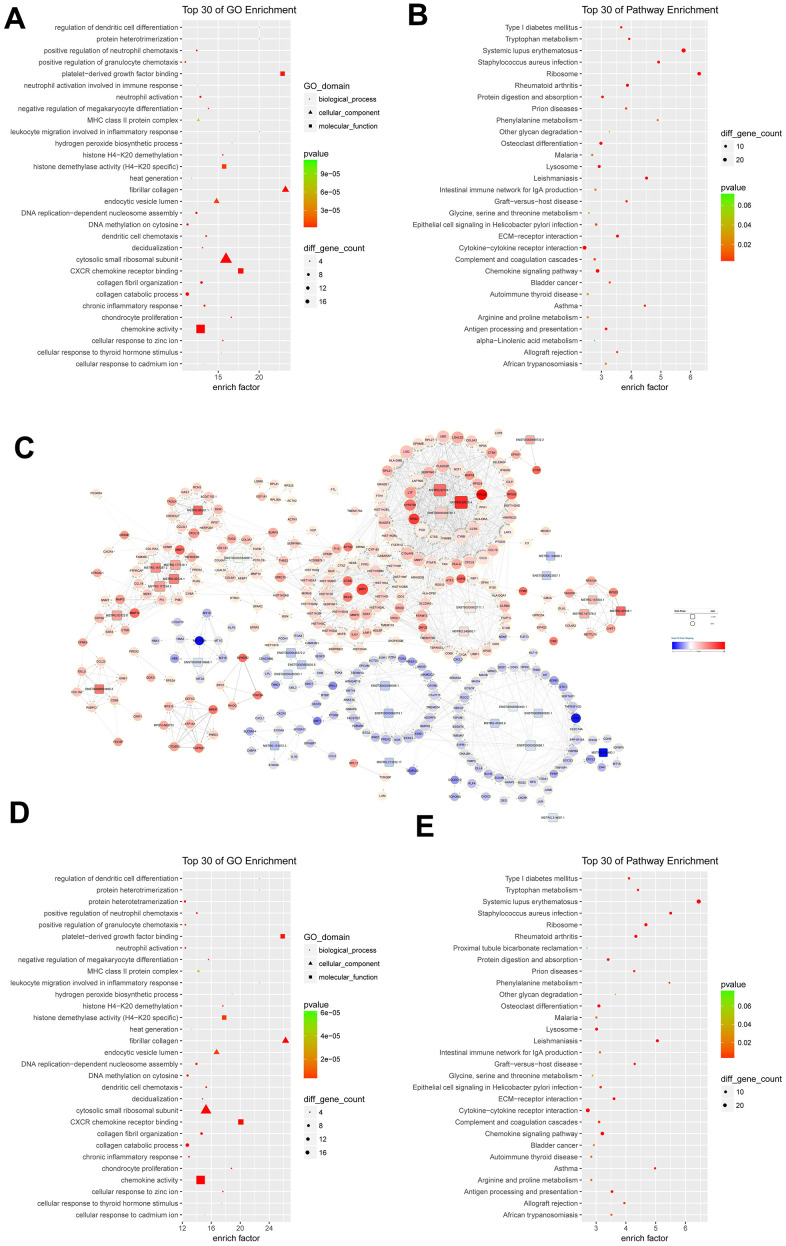
**Functional analysis of differentially expressed mRNAs and lncRNA-mRNA co-expression network.** (**A**) the top 30 Gene Ontology (GO) terms in biological process (BP), cellular component (CC), and molecular function (MF) categories for differentially expressed mRNAs; (**B**) the top 30 KEGG pathways enriched for differentially expressed mRNAs; (**C**) lncRNA-mRNA co-expression network constructed by co-expressed lncRNA-mRNA correlations; (**D**) the top 30 significant GO terms for differentially expressed mRNAs in lncRNA-mRNA co-expression network; (**E**) the top 30 significant KEGG pathways enriched for differentially expressed mRNAs in lncRNA-mRNA co-expression network; (**C**) lncRNA-mRNA co-expression network constructed by co-expressed lncRNA-mRNA correlations.

GO functional enrichment analysis indicated that differentially expressed mRNAs were significantly related to the fibrillar collagen (enrich factor = 23.22), platelet-derived growth factor binding (enrich factor = 22.87), and protein heterotrimerization (enrich factor = 20.13) (Figure 2A). Pathway enrichment analysis suggested these differentially expressed mRNAs were involved in the pathways of ribosome (enrich factor = 6.38), systemic lupus erythematosus (enrich factor = 5.87), and staphylococcus aureus infection (enrich factor = 4.99) (Figure 2B).

### LncRNA-mRNA co-expression network

The correlations between differentially expressed lncRNAs and differentially expressed mRNAs were revealed to construct the lncRNA-mRNA co-expression network. The lncRNA-mRNA co-expression network was consisted of 1956 positive correlations and 6 negative correlations involving 33 differentially expressed lncRNAs and 340 differentially expressed mRNAs. Five up-regulated lncRNAs, including *MSTRG.93125.4* (40 degrees), *ENST00000429730.1* (35 degrees), *MSTRG.93124.1* (31 degrees), *ENST00000602711.1* (21 degrees), and *MSTRG.177254.3* (17 degrees) correlated with more than 15 targeted mRNAs (Figure 2C). Regarding mRNAs, eight up-regulated mRNAs had a degree number larger than 35. They were *C15orf48* (41 degrees), *RASSF4* (38 degrees), *CCL5* (37 degrees), *CYBB* (37 degrees), *PGD* (37 degrees), *LAP3* (36 degrees), *LAPTM5* (36 degrees), and *LGI2* (36 degrees) (Figure 2C).

The differentially expressed mRNAs in lncRNA-mRNA co-expression network were enriched with the fibrillar collagen, platelet-derived growth factor binding, protein heterotrimerization, regulation of dendritic cell differentiation, and leukocyte migration involved in inflammatory response (Figure 2D). Pathway enrichment analysis for differentially expressed mRNAs in lncRNA-mRNA co-expression network indicated that the top ranked pathways were involved systemic lupus erythematosus, *Staphylococcus aureus* infection, phenylalanine metabolism, Leishmaniasis, and asthma (Figure 2E).

### Target gene prediction of differentially expressed lncRNAs and functional analysis

**Figure 3:**
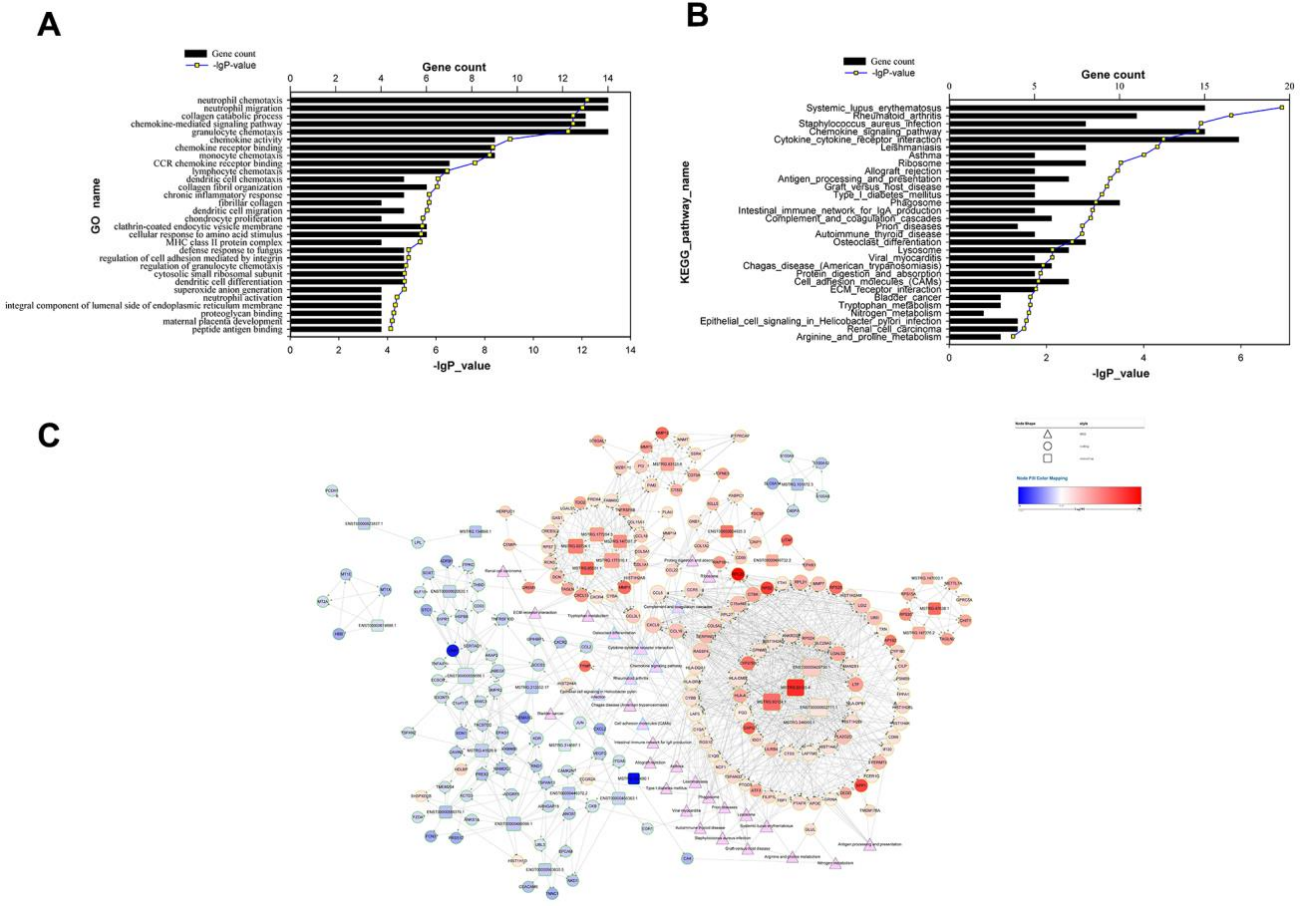
**Functional analysis of target genes predicted for differentially expressed lncRNAs and the lncRNA-target-pathway network.** (**A**) significant GO terms for differentially expressed target genes predicted for differentially expressed lncRNAs; (**B**) significant KEGG pathways enriched for differentially expressed target genes predicted for differentially expressed lncRNAs; (**C**) lncRNA-target-pathway network constructed by regulatory correlations of differentially expressed lncRNAs with predicted cis- or trans-target genes, as well as the top 30 KEGG pathways.

One cis-target gene and 440 trans-target genes were selected from the lncRNAs differentially expressed in PET-high lung tissues (See Materials and Methods). The subset of these genes that correlated significantly with the lncRNAs (|R| > 0.85 and FDR < 0.0005) were predicted as target genes and chosen for further functional and pathway enrichment analysis. All of these lncRNA target genes were differentially expressed (Supplementary Table 4). These predicted target genes were mainly involved in the biological processes of chemokine-mediated signaling pathway, collagen catabolic process, neutrophil chemotaxis, neutrophil migration, and collagen metabolic process (Figure 3A). KEGG pathway enrichment analysis indicated the predicted target gene subset were involved in the pathways of systemic lupus erythematosus, rheumatoid arthritis, *Staphylococcus aureus* infection, chemokine signaling pathway, and cytokine-cytokine receptor interaction (Figure 3B).

We then visualized the top 30 KEGG pathways, the predicted target genes, as well as the differentially expressed lncRNAs together in a lncRNA-target-pathway network map (Figure 3C). 5 lncRNAs (*ENST00000429730.1*, *MSTRG.93125.4*, *MSTRG.93124.1*, *ENST00000602711.1*, and *ENST00000555688.1*) and 9 target genes (*HLA-DMB*, *RASSF4*, *CCL5*, *CXCL9*, *C15orf48*, *CCR5*, *CYBB*, *LGI2*, and *PGD*) had a degree of number more than 25. These extensively connected genes were involved in pathways that were enriched with connections: cytokine-cytokine receptor interaction (involving *CCL5*, *CXCL9* and *CCR5*), chemokine signaling pathway (involving *CCL5*, *CXCL9* and *CCR5*), and cell adhesion molecules (involving *HLA-DMB* and *HLA-DPB1*). *RASSF4* regulates PIP2 production, a critical step for phagocytosis and phagosome formation. *C15orf48* encodes a novel subunit of the mitochondrial electron transport chain (32045714). Leucine-rich repeat glioma inactivated gene member encoded by *LGI2* plays an important role in remodeling of synapses between nerve cells (23713523). *PGD* encodes 6-phosphogluconate dehydrogenase in the pentose phosphate pathway, which converts 6-phosphogluconate into ribulose 5-P, a metabolite product that supports inflammatory macrophage responses (25904920). Other highly-connected pathways in the lncRNA-target-pathway network map included complement and coagulation cascades (involving *C1QB* and *C1QA*), lysosome (involving *CD68*, *CTSD*, and *CTSK*), phagosome (involving *CYBB* and *CYBA*). Four highly-connected target genes *CCL5*, *CXCL9*, *CYBB*, and *HLA-DMB* were predicted target genes of two highly-connected lncRNAs *ENST00000429730.1* and *MSTRG.93124.1* with 54 and 53 predicted target genes, respectively.

### Expression validation and prediction efficiencies of lncRNA indicators

**Figure 4:**
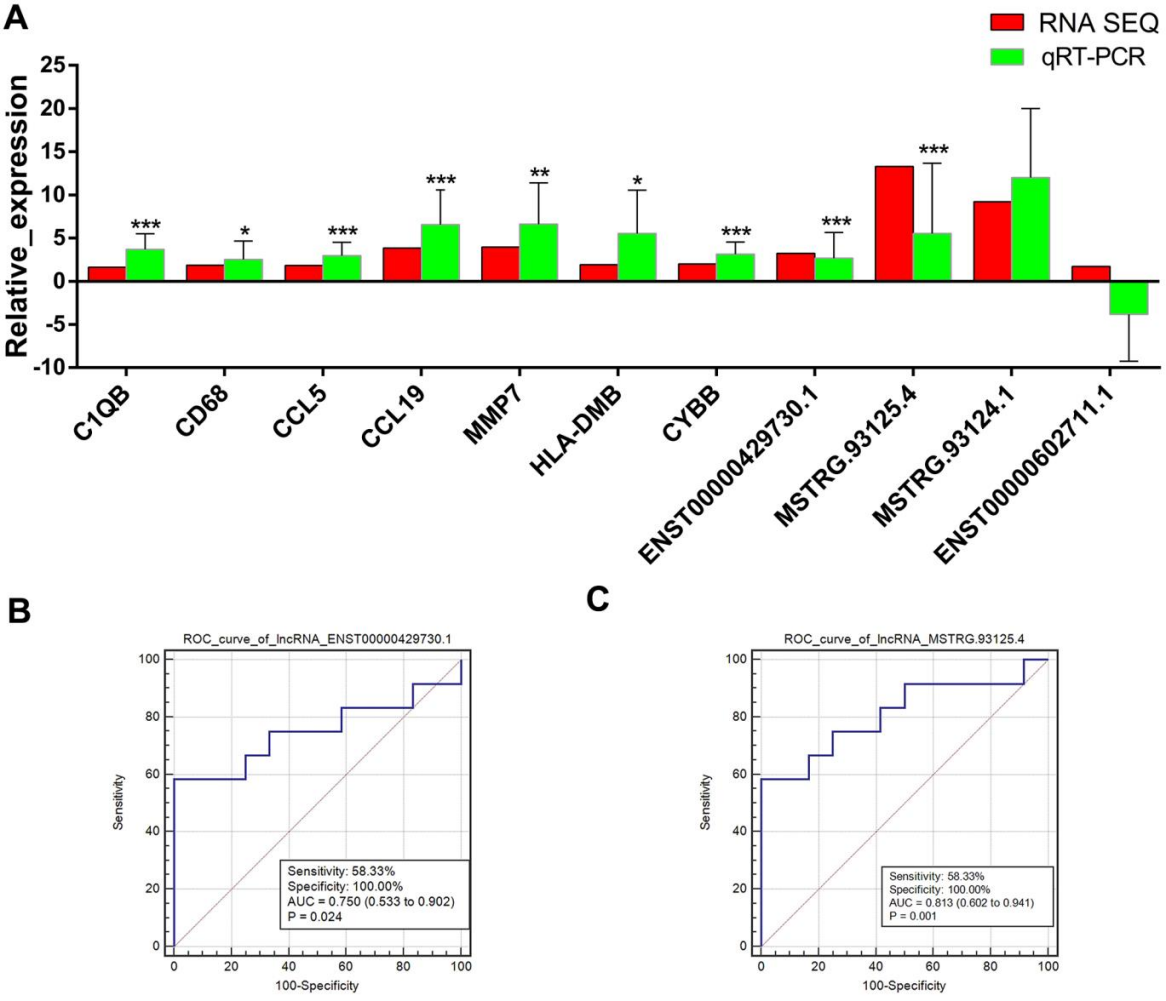
**Validation of lncRNAs and mRNAs by quantitative real-time polymerase chain reaction (qRT-PCR) and prediction efficiencies evaluated by receiver operating characteristic (ROC) curve analysis.** (**A**) expressions changes of lncRNAs and mRNAs that had relatively larger number of degree in lncRNA-target-pathway network detected by qRT-PCR. The red bars indicate the expression changes in PET-high samples comparing with PET-low samples according to the RNA-sequencing data of PET-high (n = 5) and PET-low (n = 5) samples. The green bars indicate the expression changes in PET-high samples (n = 11) comparing with PET-low samples (n = 11) according to the qRT-PCR experiment results. The horizontal axis means the names of lncRNAs or mRNAs, and the vertical axis means the relative expression (log2 FC). *: p < 0.05, **: p < 0.01, ***: p < 0.001 comparing with PET-low samples based on qRT-PCR experiment results. (**B**) ROC curve of *ENST00000429730.1*. (**C**) ROC curve of *MSTRG.93125.4*.

Expression levels of 7 mRNAs (*C1QB*, *CD68*, *CCL5*, *CCL19*, *MMP7*, *HLA-DMB*, and *CYBB*) and 4 lncRNAs (*ENST00000429730.1*, *MSTRG.93125.4*, *MSTRG.93124.1*, and *ENST00000602711.1*) that had relatively larger number of degree in lncRNA-target-pathway network were measured in the lung PET-high and PET-low tissue samples collected from 11 patients. The expression levels of *C1QB*, *CD68*, *CCL5*, *CCL19*, *MMP7*, *HLA-DMB*, and *CYBB* were validated to be significantly up-regulated in lung PET-high tissue samples (Figure 4A). Obviously higher expressions of two lncRNAs *ENST00000429730.1* and *MSTRG.93125.4* were found in lung PET-high tissue samples (Figure 4A). ROC curve analysis of *ENST00000429730.1* showed that the AUC was 0.750 with a sensitivity of 58.33% and a specificity of 100.00% (p = 0.024) (Figure 4B), and the AUC of *MSTRG.93125.4* was 0.813 with a sensitivity of 58.33% and a specificity of 100.00% (p = 0.001) (Figure 4C).

### Bioinformatics analysis of ENST00000429730.1 and MSTRG.93125.4

**Figure 5:**
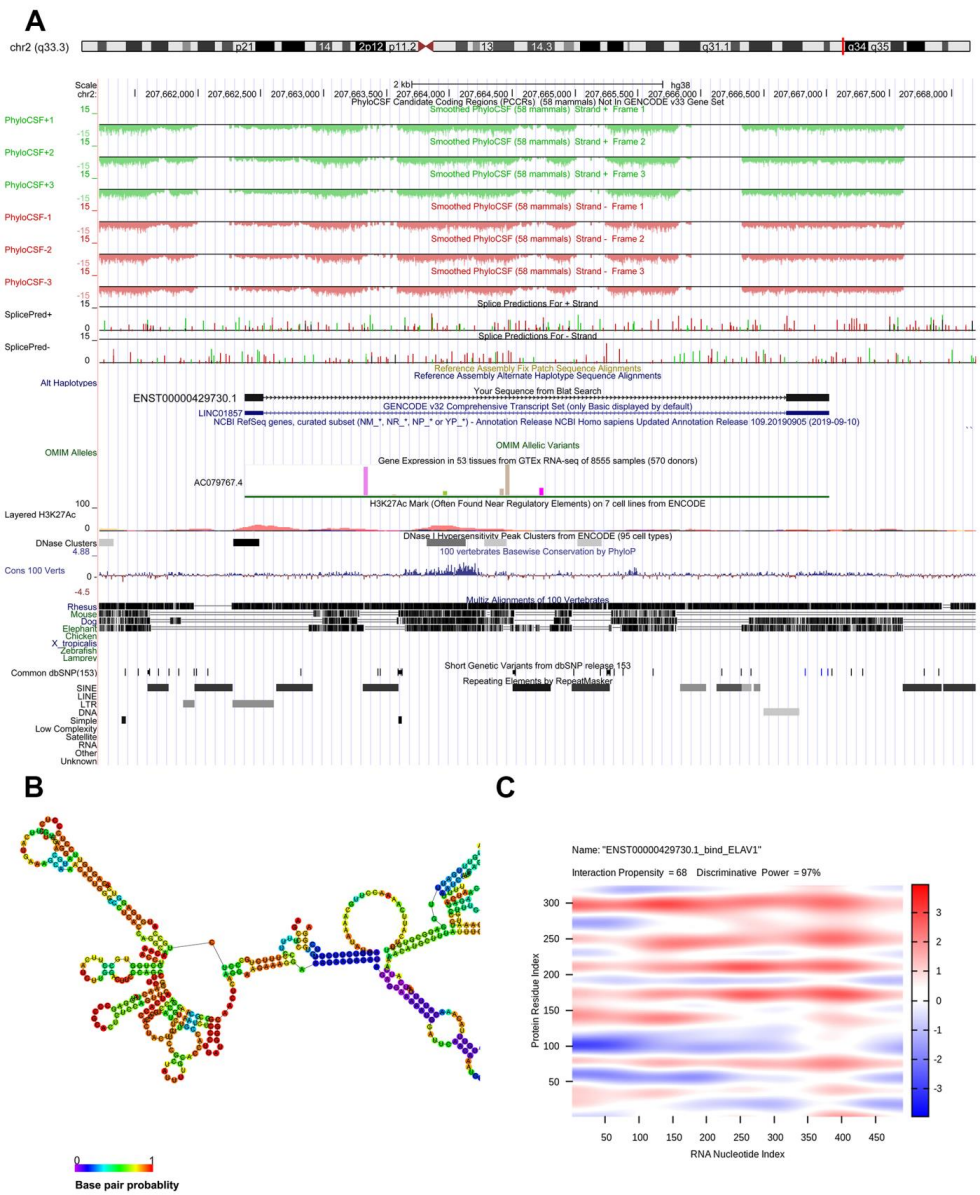
**Bioinformatics analysis of ENST00000429730.1.** (**A**) chromosome location of *ENST00000429730.1*; (**B**) optimal secondary structure for *ENST00000429730.1*; (**C**) interaction between *ENST00000429730.1* and ELAV1.

Genomic location of *ENST00000429730.1* was predicted to be on chromosome 2q33.3 and assumed to be unable to encode genes (Figure 5A). The optimal secondary structure for *ENST00000429730.1* had several hairpin loops with a minimum free energy (MFE) of -110.50 kcal/mol (Figure 5B). There were strong interactions of *ENST00000429730.1* with proteins of ELAV1, TRA2B, PTBP1, SRSF9, and SRS10. The interaction between *ENST00000429730.1* and ELAV1 has an interaction propensity of 68, and with the discriminative power of 97% (Figure 5C).

**Figure 6:**
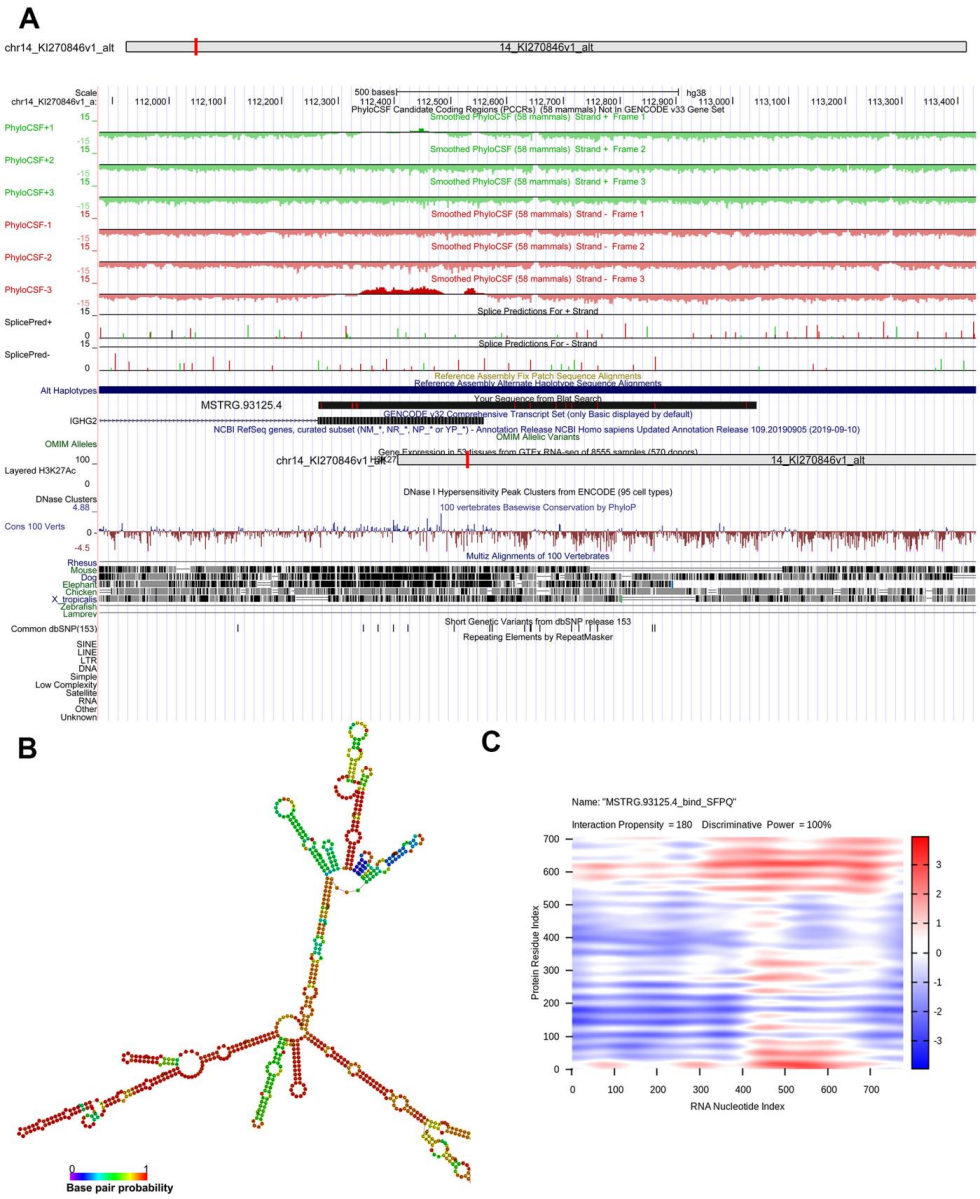
**Bioinformatics analysis of MSTRG.93125.4.** (**A**) chromosome location of *MSTRG.93125.4*; (**B**) optimal secondary structure for *MSTRG.93125.4*; (**C**) interaction between *MSTRG.93125.4* and SFPQ.

**Figure 7:**
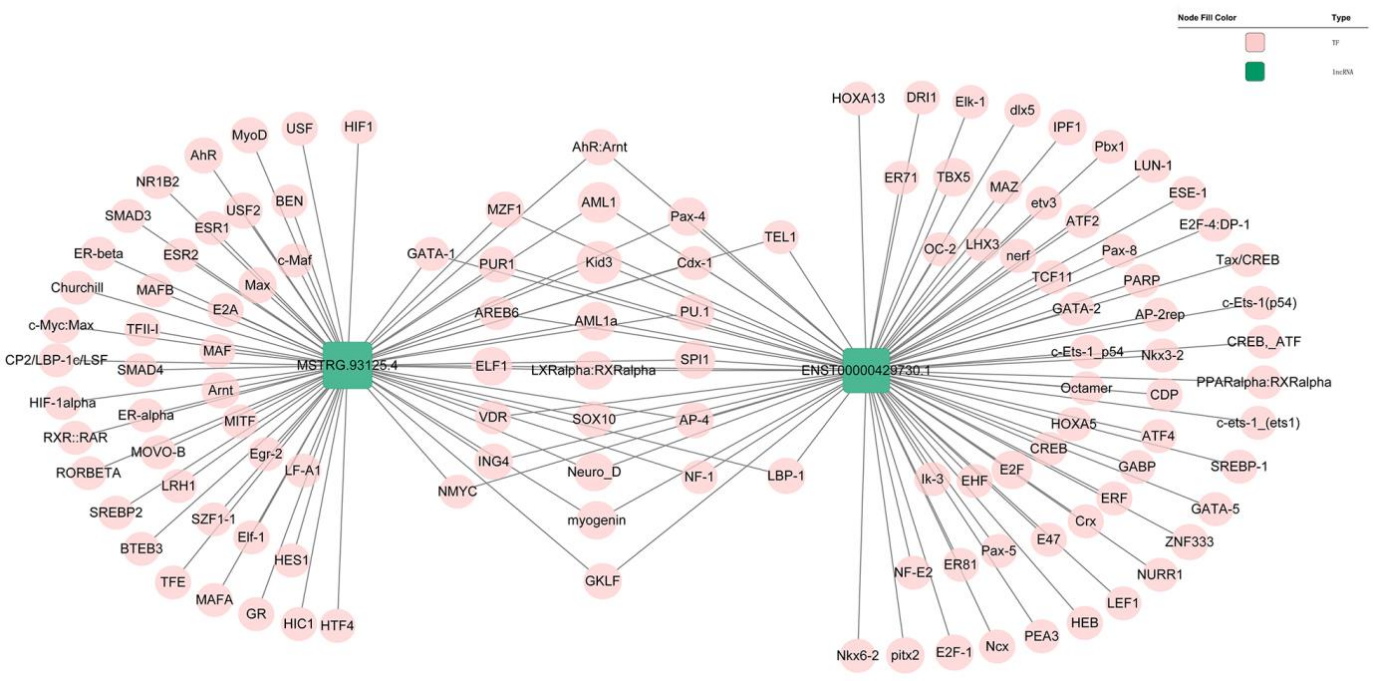
**Prediction of the potential transcriptional factors of the ENST00000429730.1 and MSTRG.93125.4.**

Genomic location of *MSTRG.93125.4* was predicted to be on chromosome 14KI270846v1_alt and unable to encode genes (Figure 6A). The optimal secondary structure for *MSTRG.93125.4* had the MFE of -359.20 kcal/mol (Figure 6B). For *MSTRG.93125.4*, strong interactions were found with proteins of SFPQ, PCBP1, LN28B, SRSF2, and PCBP2. The interaction between *MSTRG.93125.4* and SFPQ has an interaction propensity of 180, with the discriminative power of 100% (Figure 6C). Prediction analysis of potential transcriptional factors showed that *MSTRG.93125.4* can combine with 67 transcriptional factors (such as Kid3, AML1, myogenin, MyoD, and AP-4), and *ENST00000429730.1* can combine with 82 transcriptional factors (such as *AML1*, *TBX5*, *E2F*, *IPF1*, and E2F-1) (Figure 7).

## DISCUSSION

Tuberculosis, an infectious disease caused by *M. tuberculosis*, remains a major cause of ill health burden and is one of the leading causes of death globally [2]. Although detection techniques and therapeutic approaches for tuberculosis have achieved significant developments, there is still no suitable indicators for complete cure of tuberculosis in patients with sputum-negative tuberculosis. In our study, 386 differentially expressed mRNAs and 44 differentially expressed lncRNAs were identified in lung PET-high tissue samples according to the RNA-sequencing profiles. The differentially expressed mRNAs in lncRNA-mRNA co-expression network were notably associated with the fibrillar collagen, platelet-derived growth factor binding, protein heterotrimerization, regulation of dendritic cell differentiation, and leukocyte migration involved in inflammatory response.

Tuberculosis is characterized by chronic inflammation and tissue fibrosis. Fibrillar collagens, determinants of lung tensile strength and highly resistant to enzymatic degradation, play important roles in lung injury and repair. Only collagenolytic matrix metalloproteinases (MMPs) have unique abilities to cleave these helical collagens at neutral pH [18, 19]. It has been reported that *M. tuberculosis* infection increased MMP-1 expression, resulting in significantly greater collagen breakdown and alveolar destruction in lung granulomas [20]. Interferon-γ released from lymphocytes stimulated by the *M. tuberculosis* antigen resulted in increased platelet-derived growth factor-B (PDGF-B) in alveolar macrophages, suggesting a link between the delayed type hypersensitivity response and the first stages of a fibrotic reaction in the lungs of patients with untreated pulmonary tuberculosis [21, 22]. Thus, differentially expressed lncRNAs may co-expressed with differentially expressed mRNAs to influence the functions of fibrillar collagen, platelet-derived growth factor binding, as well as leukocyte migration involved in inflammatory response in lung PET-high tissue samples.

Afterwards, one cis-target gene and 440 trans-target genes were predicted for differentially expressed lncRNAs. The expression levels of *C1QB*, *CD68*, *CCL5*, *CCL19*, *MMP7*, *HLA-DMB*, and *CYBB* were validated to be significantly elevated in lung PET-high tissue samples. and Obviously higher expressions of two lncRNAs *ENST00000429730.1* and *MSTRG.93125.4* were found in lung PET-high tissue samples. What’s more, *CCL5*, *CXCL9*, *CYBB*, and *HLA-DMB* were the predicated trans-target genes *ENST00000429730.1* and *MSTRG.93125.4*. *CCL5* and *CCL19* were involved in the cytokine-cytokine receptor interaction and chemokine signaling pathway. Cytokines and chemokines play important roles in the initiation, sequential recruitment and activation of cells into *M. tuberculosis* infected lungs [23]. In the mouse model of *M. tuberculosis* infection, three CCR5 ligands *CCL3*, *CCL4*, *and CCL5* are overexpressed in the lungs, among which *CCL5* being induced to the highest level. When *CCL5* gene deficient mice were infected with *M. tuberculosis,* transient early impairment in granuloma formation and delayed T cell recruitment were observed [24, 25]. *CCR7* ligation by *CCL19* and *CCL21* is vital for the positioning of T cells and dendritic cells of secondary lymphoid tissues, and *CCR7*-deficient mice, which are deficient in *CCL19* and *CCL21* signaling, are fully capable to control pulmonary tuberculosis [26, 27]. Meanwhile, differentially expressed target genes were significant enriched in phagosome (such as *CYBB*) and cell adhesion molecules (such as *HLA-DMB*), which were used to construct the lncRNA-target-pathway network. Congenital X-linked mutations in *CYBB*, which encoding the gp91 (phox) subunit of the phagocyte NADPH oxidase, could lead to recurrent tuberculosis in patients with defects preferentially manifest in macrophages, suggesting *CYBB* is associated with mendelian susceptibility to mycobacterial disease and the respiratory burst in human macrophages is a crucial mechanism for protective immunity to tuberculous mycobacteria [28, 29]. Therefore, differentially expressed lncRNAs may influence the cytokine-cytokine receptor interaction, chemokine signaling pathway, phagosome, and cell adhesion molecules by regulating their target genes in lung PET-high tissue samples.

Prediction efficiency analysis showed that the AUC of *ENST00000429730.1* and *MSTRG.93125.4* was 0.750 and 0.813 respectively. These two lncRNAs might be the potential biomarkers for active tuberculosis. Strong interactions of *ENST00000429730.1* with ELAV1, and *MSTRG.93125.4* with SFPQ were revealed. Solute carrier family 11 member 1 (*SLC11A1*) has been reported to be involved in susceptibility to human immunodeficiency virus and tuberculosis infection, and RNA binding protein HuR (ELAV1) is a key mediator of stabilization of *SLC11A1* mRNA and SLC11A1 protein expression [30, 31]. Splicing factor proline- and glutamine-rich (SFPQ) protein, also known as polypyrimidine tract binding protein-associated splicing factor (PSF), is a multifunctional nuclear protein that participates in cellular activities, such as RNA transport, DNA repair, and apoptosis [32]. Secreted *M. tuberculosis* Rv3654c protein participate in the suppression of macrophage apoptosis by recognizing and interacting with PSF to cleave it, diminishing the availability of caspase-8, and inactivation of PSF also significantly decreased the level of caspase-8 in macrophages [33, 34]. Taken together, interactions of *ENST00000429730.1* with ELAV1, and *MSTRG.93125.4* with SFPQ might influence the metabolic activity in tuberculosis lesions. However, the specific regulatory mechanisms related to interactions of *ENST00000429730.1* with ELAV1, and *MSTRG.93125.4* with SFPQ in the metabolic activity of tuberculosis lesions need to be explored by further studies.

In conclusion, analysis of RNA-sequencing profiles identified 386 differentially expressed mRNAs and 44 differentially expressed lncRNAs in the PET-high lung samples. Two lncRNAs *ENST00000429730.1* and *MSTRG.93125.4* might be considered as potential biomarkers of metabolic activity in tuberculosis lesions for patients with sputum-negative tuberculosis. Meanwhile, *ENST00000429730.1* and *MSTRG.93125.4* may influence the cytokine-cytokine receptor interaction, chemokine signaling pathway, phagosome, and cell adhesion molecules by regulating their trans-target genes, such as *CCL5*, *CXCL9*, *CYBB*, and *HLA-DMB*. Moreover, interactions of *ENST00000429730.1* with ELAV1, and *MSTRG.93125.4* with SFPQ might associated with metabolic activity in tuberculosis lesions.

## MATERIALS AND METHODS

### Patients and samples

**Figure 8:**
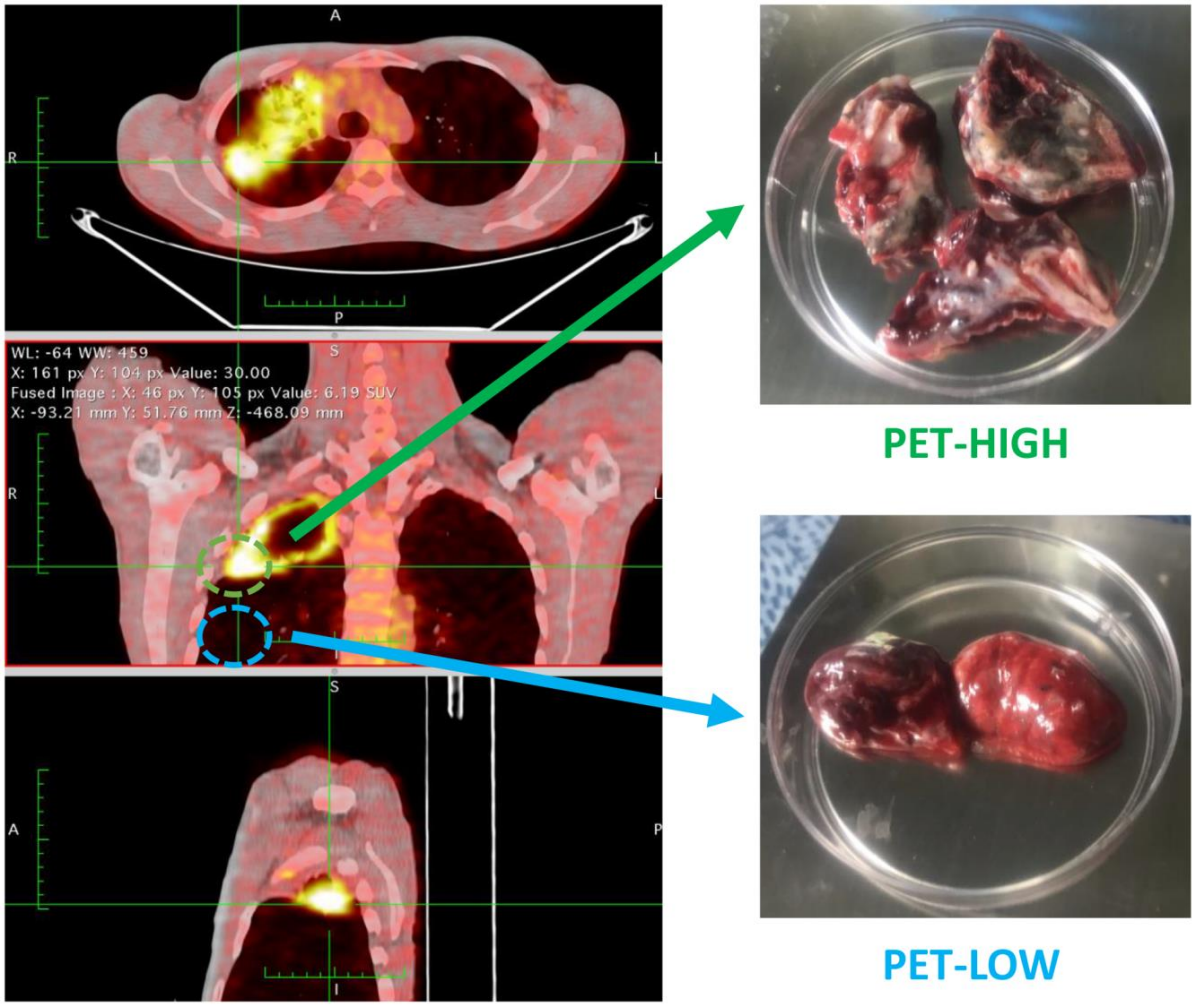
**Lung tissue samples with high metabolic activity (PET-high) and low metabolic activity (PET-low) demonstrated by fluorine-18-fluorodeoxyglucose positron emission tomography combined with computed tomography (FDG-PET/CT) were collected for RNA-sequencing.**

Five patients who diagnosed as sputum-negative pulmonary tuberculosis in our hospital from June 06 to December 2018 were enrolled for RNA-sequencing analysis. There were one male and four females, aged 20-38 years, with an average age of 31.2 ± 7.4 years (Supplementary Table 1). Lung tissue samples with high metabolic activity (PET-high) and low metabolic activity (PET-low) demonstrated by (FDG-PET/CT) were collected from these five participants for the subsequent sequencing (Figure 8). Moreover, lung PET-high and PET-low tissue samples collected from 11 enrolled subjects were used for experimental validation of expression changes by quantitative real-time PCR (qRT-PCR). There were 7 male and 4 females, aged 20-57 years, with an average age of 38.3 ± 11.3 years (Supplementary Table 2).

All the subjects were confirmed as pulmonary tuberculosis by pathological examination with sputum smear and culture negative preoperatively, and accompanied by tuberculous pleurisy or other extrapulmonary tuberculosis. The included participants were demonstrated to have residual metabolic activity by FDG-PET/CT, and in non-human immunodeficiency virus (non-HIV) infection status. Patients were excluded when they met one of the following criteria: sputum positive for mycobacterium tuberculosis complex by Xpert or culture (bacteriologically confirmed tuberculosis disease) before surgery; in the presence of tuberculosis symptoms; signs or symptoms of acute illness; with the diagnosis of malignancy; any clinical condition requiring systemic steroid or other immunosuppressive medication in the preceding six months; uncontrolled diabetes mellitus, pregnant or breastfeeding; anemia (hemoglobin < 7g/dL); significant smoking history (> 30 pack-years). All patients signed informed consent to participate in the study. This study was approved by the Ethics Committee of the Shanghai Public Health Clinical Center.

### RNA-sequencing by Illumina HiSeq

Total RNA of each lung sample was extracted by using TRIzol Reagent (Invitrogen, US) and further purified using the RNeasy Mini Kit (Qiagen, Germany) according to the manufacturer’s instructions. Total RNA of each sample was quantified and qualified by Agilent 2100 Bioanalyzer (Agilent Technologies, USA), NanoDrop (Thermo Fisher Scientific Inc., USA) and 1% agarose gel. Total RNA (1 μg) with the value of RNA integrity number (RIN) > 7 was used for library preparation of the following next generation sequencing, with the NEBNext Ultra II Directional RNA Library Prep. Kit for Illumina. The rRNA was depleted from total RNA using Ribo-Zero™ rRNA removal Kit (Illumina, USA), and cDNA libraries were generated following manufacture’s protocols. Then, libraries with different indices were multiplexed and loaded on Illumina HiSeq instrument (Illumina, USA) for 2 × 150 paired-end sequencing according to manufacturer’s instructions in the Medical Laboratory of Nantong ZhongKe.

### RNA-sequencing data analysis

Generated raw reads were saved as fastq format. Raw reads were processed by Trimmomatic (version 0.30) to filter dirty reads that with sequence adaptors, primers, fragments, or length of bases less than 20. Afterwards, high quality clean data were aligned to reference genome via software Hisat2 (version 2.0.1). Comparing with the PET-low samples, the mRNAs differentially expressed in PET-high samples were uncovered by the DESeq Bioconductor package. The cut-off criteria were set as |log_2_ fold change (FC) | > 1 and q value < 0.05, after adjusted by Benjamini and Hochberg’s approach. Hierarchical clustering analysis based on centered Pearson correlation was performed for all the differentially expressed mRNAs via the pheatmap (version 1.0.8) [35, 36]. Meanwhile, differentially expressed lncRNAs between PET-high and PET-low samples were identified by the DESeq Bioconductor package, with the thresholds of |log_2_ FC| > 0.5 and false discovery rate (FDR) < 0.05.

In order to identify functions and specific signaling pathways of differentially expressed genes, Gene Ontology (GO) and KEGG pathway analyses were performed. The thresholds were set as gene number ≥ 5 and enrichment test p-value ≤ 0.01 [37, 38].

### Construction of lncRNA-mRNA co-expression network

The correlations of differentially expressed lncRNAs with differentially expressed mRNAs were evaluated by calculating the Pearson correlation coefficient R values. Fisher's asymptotic test implemented in the WGCNA library of R was adopted to estimate p-values for each correlation pair. The p-value was further adjusted into FDR by Bonferroni multiple test correction implemented in the multtest package of R [39]. The lncRNA-mRNA correlations with the |R| > 0.9 and FDR < 0.005 were regarded as co-expressed and used to construct lncRNA-mRNA co-expression network. The co-expression network was visualized by Cytoscape software. GO functional and KEGG pathway enrichment analysis were conducted for the differentially expressed mRNAs in the lncRNA-mRNA co-expression network, with the criterion of p < 0.05.

### LncRNA target prediction and regulatory network construction

Target genes of differentially expressed lncRNAs were predicted via cis- or trans-regulatory effects. LncRNAs and potential target genes were paired and visualized using University of California Santa Cruz (UCSC) genome browser (http://genome-asia.ucsc.edu/index.html). Genes transcribed within a 10 kbp window upstream or downstream of lncRNAs and co-expressed with the lncRNA genes were considered as cis target genes using Bedtools (version 2.17.0). The algorithm for regulation in trans is based on mRNA sequence complementarity and then RNA duplex energy prediction. First, BLAST (version 2.2.28+) software was used sequence complementarity. RNAplex was then used to select trans-acting target gene with threshold parameter of duplex energy–e set as 20.

KEGG pathway analysis was performed for the predicted cis- or trans-target genes among differentially expressed genes to identify important signaling pathways. The correlations of differentially expressed lncRNAs with predicted cis- or trans-target genes were evaluated by calculating the Pearson correlation coefficient R values. The lncRNA-target gene correlations with the |R| > 0.85 and FDR < 0.0005, and the top 30 KEGG pathways were used to construct lncRNA-target pathway network.

### Validation of lncRNA and mRNA expressions

The expressions of lncRNAs and mRNAs that had high number of degree in lncRNA-target-pathway network were further confirmed by qRT-PCR. Lung PET-high and PET-low tissues were collected from 11 patients. Total RNA was isolated from each sample using TRIzol reagent and treated with DNase I (Invitrogen) according to the manufacturer’s protocol. RNA was reverse transcribed into cDNA using the PrimeScript RT reagent Kit (Takara, Shiga, Japan) and amplified using the SYBR Green Kit (Takara, Shiga, Japan) on an ABI Prism 7300 sequence detection system (Applied Biosystems, Foster City, USA). The reaction mixture contained cDNA, 200 nM of each primer and 10 μl of 2 × SYBR Green PCR Master Mix (Takara, Shiga, Japan). The primer sequences of lncRNAs and mRNAs were listed in Supplementary Table 3. Reactions were incubated at 95° C for 30s, followed by 40 cycles of 95° C for 10 s and 60° C for 30 s. Reactions were run in triplicate for analysis. At the end of the PCR cycles, melting curve analysis was performed to verify product specificity. Expression levels of lncRNAs and mRNAs were normalized to housekeeping gene GAPDH and the 2^-ΔΔCt^ method was applied to calculate the relative expression levels.

### Prediction efficiencies and bioinformatics analysis of lncRNA-binding proteins

The lncRNAs validated by qRT-PCR were further remained as indicators for lesion with high metabolic activity. The prediction efficiencies of lncRNAs were evaluated by receiver operating characteristic (ROC) curve analysis via pROC package (version 1.14.0) to calculate the area under the curve (AUC) [40].

Genomic locations of lncRNA-binding proteins were predicted by using UCSC Genome Browser (http://genome-asia.ucsc.edu/index.html). Secondary structures were identified via RNAfold minimum free energy estimations based on RNAfold webserver (http://rna.tbi.univie.ac.at/cgi-bin/RNAWebSuite/RNAfold.cgi). TRANSFAC (http://www.gene-regulation.com/index2.html) was used to predict the potential transcription factors (TFs) of lncRNAs. Moreover, catRAPID analysis (http://service.tartaglialab.com/) was conducted to predict the potential interacting proteins of lncRNAs.

### Ethical approval

The Permission of the Hospital Ethics Committee was obtained by the Shanghai public health clinical center's ethics committee before operation for each patient. (Approval number:2019-S009-02).

## Supplementary Material

Supplementary Tables 1, 2 and 3

Supplementary Table 4
