# Supplementary material for: Long non-coding RNAs ENST00000429730.1 and MSTRG.93125.4 are associated with metabolic activity in tuberculosis lesions of sputum-negative tuberculosis patients
Source: Aging (Albany NY). 2021 Mar 3;13(6):8228–47. doi: 10.18632/aging.202634 (PMC8034958; doi:10.18632/aging.202634)
Supplement: Supplementary Tables 1, 2 and 3 [file aging-13-202634-s001.pdf]

SUPPLEMENTARY TABLES

Supplementary Table 1. The clinical information about the 5 patients whose lung tissue samples with high metabolic activity and low metabolic activity were collected for RNA-sequencing.

Patients No.	Gender	Age (Year)	Clinical diagnosis	History of tuberculosis treatment	Preoperative sputum smear and culture	Nucleic acid PCR	Preoperative Xpert	Maximum SUV from PET/CT	Postoperative pus smear and culture	Postoperative pus Xpert	Detection of drug resistance genes	Postoperative pathology
1	Male	38	Pulmonary tuberculosis	The first treatment lasted for 6 months, and recurrence was observed one year after drug withdrawal, followed by treatments for more than 4 years	Negative	Negative	Negative	6.19	Negative	Negative	Negative	Epithelioid granuloma formation, negative by acid fast staining, and coagulative necrosis
2	Female	33	Pulmonary tuberculosis, and spinal tuberculosis	The first treatment lasted for 12 months, and cervical lymphoid tuberculosis was found 2 months after drug withdrawal, followed by treatments for more than 2 years	Negative	Negative	Negative	3.87	Negative	Negative	Mycobacterium tuberculosis (first-line drug sensitive)	Epithelioid granuloma formation, negative by acid fast staining, and completely coagulative necrosis
3	Female	37	Multi-drug resistant tuberculosis, and left side chest wall tuberculosis with sinus formation	More than 4 months of anti-tuberculosis treatment	Negative	Negative	Negative	4.8	1+	Negative	Rifampin and isoniazide resistance	Granuloma formation, complete coagulative necrosis, and 7 positive bacteria were found by acid fast staining
4	Female	28	Secondary pulmonary tuberculosis with multi-drug resistance	More than 20 months of anti-tuberculosis treatment	Negative	Negative	Negative	3.84	Negative	Negative	Rifampin (S531L), ethambutol (306M2), streptomycin (43M), and isoniazid (315M) resistance site mutation	Epithelioid granuloma formation, negative by acid fast staining, and completely coagulative necrosis
5	Female	20	Pulmonary tuberculosis, and tuberculous pleurisy	More than 4 months of anti-tuberculosis treatment	Negative	Negative	Negative	10	Negative	Negative	Mycobacterium tuberculosis (first-line drug sensitive)	Coagulative necrosis and 4 positive bacteria were found by acid fast staining

PET/CT: Positron Emission Tomography/Computed Tomography; SUV: standard uptake value.

Supplementary Table 2. The clinical information about the 11 patients whose lung tissue samples with high metabolic activity and low metabolic activity were collected for experimental validation.

Patients No.	Gender	Age (Year)	Clinical diagnosis	History of tuberculosis treatment	Preoperative sputum smear and culture	Nucleic acid PCR	Preoperative Xpert	Maximum SUV from PET/CT	Postoperative pus smear and culture	Postoperative pus Xpert	Detection of drug resistance genes	Postoperative pathology
1	Male	38	Pulmonary tuberculosis	The first treatment lasted for 6 months, and recurrence was observed one year after drug withdrawal, followed by treatments for more than 4 years	Negative	Negative	Negative	6.19	Negative	Negative	Negative	Epithelioid granuloma formation, negative by acid fast staining, and coagulative necrosis
2	Female	33	Pulmonary tuberculosis, and spinal tuberculosis	The first treatment lasted for 12 months, and cervical lymphoid tuberculosis was found 2 months after drug withdrawal, followed by treatments for more than 2 years	Negative	Negative	Negative	3.87	Negative	Negative	Mycobacterium tuberculosis (first-line drug sensitive)	Epithelioid granuloma formation, negative by acid fast staining, and completely coagulative necrosis
3	Female	37	Multi-drug resistant tuberculosis, and left side chest wall tuberculosis with sinus formation	More than 4 months of anti-tuberculosis treatment	Negative	Negative	Negative	4.8	1+	Negative	Rifampin and isoniazide resistance	Granuloma formation, complete coagulative necrosis, and 7 positive bacteria were found by acid fast staining
4	Female	28	Secondary pulmonary tuberculosis with multi-drug resistance	More than 20 months of anti-tuberculosis treatment	Negative	Negative	Negative	3.84	Negative	Negative	Rifampin (S531L), ethambutol (306M2), streptomycin (43M), and isoniazid (315M) resistance site mutation	Epithelioid granuloma formation, negative by acid fast staining, and completely coagulative necrosis
5	Female	20	Pulmonary tuberculosis, and tuberculous pleurisy	More than 4 months of anti-tuberculosis treatment	Negative	Negative	Negative	10	Negative	Negative	Mycobacterium tuberculosis (first-line drug sensitive)	Coagulative necrosis and 4 positive bacteria were found by acid fast staining
6	Male	39	Obsolete pulmonary tuberculosis with Aspergillus infection	More than 2 months of anti-tuberculosis treatment	Negative	Negative	Negative	3.1	Negative	Negative	Negative	Inflammatory cell infiltration, inflammatory necrosis, interstitial hemorrhage, a

												large number of fungal spores and hyphae, and negative by acid fast staining
7	Male	28	Secondary pulmonary tuberculosis	More than 14 months of anti-tuberculosis treatment, thereafter more than 5 months of treatment for pulmonary cavity formation	Negative	Negative	Negative	4.2	Negative	Negative	Negative	Inflammatory cell infiltration in the upper left lung, epithelioid granuloma, coagulative necrosis, negative by acid fast staining, and negative by Periodic Acid-Schiff staining
8	Male	55	Cavitary pulmonary tuberculosis	More than 3 months of anti-tuberculosis treatment	2+	Negative	Negative	3.6	Positive, MPB64 negative	Negative	M.intracellulare	Inflammatory cell infiltration in the upper right lung, epithelioid granuloma, complete coagulative necrosis, and positive by acid fast staining
9	Male	57	Pulmonary tuberculosis, and spinal tuberculosis	More than 2 months of anti-tuberculosis treatment	1+	Negative	Negative	5.5	Negative	Rifampicin sensitive	Mycobacterium tuberculosis(first-line and second -line drug sensitive)	Inflammatory cell infiltration, epithelioid granuloma, and complete coagulative necrosis
10	Male	39	Pulmonary tuberculosis	More than 36 months of anti-tuberculosis treatment, followed by pulmonary cavity formation	Negative	Negative	Negative	3.6	Negative	Rifampicin sensitive	Negative	Inflammatory cell infiltration in the upper left lung, epithelioid granuloma, complete coagulative necrosis, and calcification.
11	Male	47	Pulmonary tuberculosis	More than 15 months of anti-tuberculosis treatment	Negative	Negative	Negative	11	1+	Rifampicin sensitive	Negative	Inflammatory cell infiltration in the upper left lung, epithelioid granuloma, and complete coagulative necrosis

Supplementary Table 3. Primer sequences for quantitative real-time polymerase chain reaction (qRT-PCR) analysis.

Name	Primer	Sequence (5' -> 3')	Length	Tm	Location	Amplicon Size
C1QB (NM_000491)	Forward Primer	AGGTGAATCGGGAGACTACAA	21	60.0	330-350	
	Reverse Primer	CACTGCGGGGCTCATAATTG	20	61.2	472-453	143
CD68 (NM_001251)	Forward Primer	CTTCTCTCATTCCCCTATGGACA	23	60.6	643-665	
	Reverse Primer	GAAGGACACATTGTACTCCACC	22	60.3	747-726	105
CCL5 (NM_002985)	Forward primer	TCCTCATTGCTACTGCCCTC	20	59.17	87-106	
	Reverse primer	TCGGGTGACAAAGACGACTG	20	59.97	237-256	170
CCL19 (NM_006274)	Forward Primer	TACATCGTGAGGAACTTCCACT	22	60.5	121-142	
	Reverse Primer	CTGGATGATGCGTTCTACCCA	21	61.6	252-232	132
MMP7 (NM_002423)	Forward Primer	GAGTGAGCTACAGTGGGAACA	21	61.1	81-101	
	Reverse Primer	CTATGACGCGGGAGTTTAACAT	22	60.2	238-217	158
HLA-DMB (NM_002118)	Forward Primer	ACCTGTCTGTTGGATGATGCT	21	61.1	82-102	
	Reverse Primer	CGCAAGGGGCCATCTTATTCT	21	62.2	193-173	112
CYBB (NM_000397)	Forward Primer	ACCGGGTTTATGATATTCCACCT	23	60.9	89-111	
	Reverse Primer	GATTTCGACAGACTGGCAAGA	21	60.0	223-203	135
MSTRG.93125.4 (IGHG3)	Forward primer	TGCAGGTGTAGGTCTTCGTG	20	59.68	45-64	
	Reverse primer	GTGACGGTGTCGTGGAACTC	20	60.94	167-186	142
MSTRG.93124.1 (IGHG4)	Forward primer	CTGACCTGGTTCTTGGTCATCT	22	60.5	156-177	
	Reverse primer	GAGTACAAGTGCAAGGTCTCCA	22	59.4	136-257	102
ENST00000429730.1 (LINC01857(NR_135567))	Forward primer	CCCAGCATCCCTACTCTTGC	20	59.6	125-144	
	Reverse primer	CACAGCAGTAGTCAGAAAGAACAC	24	58.4	249-272	149
GAPDH	Forward primer	ATCACTGCCACCCAGAAGAC	20	59.9	709-728	
	Reverse primer	ATGAGGTCCACCACCCTGTT	20	60.6	1125-1234	525
